# Activation of Cell Surface Bound 20S Proteasome Inhibits Vascular Cell Growth and Arteriogenesis

**DOI:** 10.1155/2015/719316

**Published:** 2015-06-04

**Authors:** Wulf D. Ito, Natalie Lund, Ziyang Zhang, Friedrich Buck, Heinrich Lellek, Andrea Horst, Hans-Günther Machens, Heribert Schunkert, Wolfgang Schaper, Thomas Meinertz

**Affiliations:** ^1^Medical Department II, Experimental Angiology, University Hospital Lübeck, Ratzeburger Allee 160, 23538 Lübeck, Germany; ^2^Department of Cardiology, University Hospital Hamburg Eppendorf, Martinistraße 52, 20246 Hamburg, Germany; ^3^Max-Planck Institute of Heart and Lung Research, Ludwigstraße 43, 61231 Bad Nauheim, Germany; ^4^Cardiovascular Center Oberallgaeu-Kempten, Academic Teaching Hospital, University of Ulm, Im Stillen 2, 87509 Immenstadt, Germany; ^5^Department of Orthopedics, Hand Surgery Division, Tongji Hospital, Tongji Medical College, Huazhong University of Science and Technology, Wuhan, Hubei, China; ^6^Department of Plastic Surgery and Hand Surgery, Klinikum Rechts der Isar, Technical University of Munich, Ismaninger Straße 22, 81675 Munich, Germany; ^7^Department of Clinical Chemistry, University Hospital Hamburg Eppendorf, Martinistraße 52, 20246 Hamburg, Germany; ^8^Department of Medicine, University Hospital Hamburg Eppendorf, Martinistraße 52, 20246 Hamburg, Germany; ^9^Institute of Experimental Immunology and Hepatology, University Medical Center Hamburg-Eppendorf, Martinistraße 52, 20246 Hamburg, Germany; ^10^Department of Cardiology, German Heart Center Munich, Technical University of Munich, and Deutsches Zentrum für Herz-Kreislauf-Forschung (DZHK), Standard Munich Heart Alliance, Lazarettstraße 36, 80636 Munich, Germany

## Abstract

Arteriogenesis is an inflammatory process associated with rapid cellular changes involving vascular resident endothelial progenitor cells (VR-EPCs). Extracellular cell surface bound 20S proteasome has been implicated to play an important role in inflammatory processes. In our search for antigens initially regulated during collateral growth mAb CTA 157-2 was generated against membrane fractions of growing collateral vessels. CTA 157-2 stained endothelium of growing collateral vessels and the cell surface of VR-EPCs. CTA 157-2 bound a protein complex (760 kDa) that was identified as 26 kDa *α*7 and 21 kDa *β*3 subunit of 20S proteasome in mass spectrometry. Furthermore we demonstrated specific staining of 20S proteasome after immunoprecipitation of VR-EPC membrane extract with CTA 157-2 sepharose beads. Functionally, CTA 157-2 enhanced concentration dependently AMC (7-amino-4-methylcoumarin) cleavage from LLVY (*N*-Succinyl-Leu-Leu-Val-Tyr) by recombinant 20S proteasome as well as proteasomal activity in VR-EPC extracts. Proliferation of VR-EPCs (BrdU incorporation) was reduced by CTA 157-2. Infusion of the antibody into the collateral circulation reduced number of collateral arteries, collateral proliferation, and collateral conductance *in vivo.* In conclusion our results indicate that extracellular cell surface bound 20S proteasome influences VR-EPC function *in vitro* and collateral growth *in vivo.*

## 1. Introduction

Vascular plasticity as seen during angiogenesis and arteriogenesis (the growth of collateral arteries from preexisting arteriolar anastomoses) requires rapid adaptation of endothelial and smooth muscle cells to environmental changes such as hypoxia and shear force [1–3]. Thus on-site protein turnover is of exquisite importance. The ubiquitin-proteasome pathway is involved in most regulatory mechanisms in which such a timely response is required, presumably because it offers extremely high substrate specificity and the ability to alter quickly the rate of proteolysis [4]. One example is the regulation of hypoxia inducible transcription factor HIF 1 alpha that plays an important role during angiogenesis [5–7].

While oxygen tension plays a paramount role during angiogenesis the growth of preexisting arteriolar or arterial anastomoses into collateral arteries (arteriogenesis) is mainly driven by local changes of hemodynamical forces, which lead to rapid adaptations of arteriolar anastomoses via pronounced morphological changes [1, 2, 8]. Maximal proliferation is observed during the first three days after induction of a hemodynamically relevant stenosis and is associated with a pronounced accumulation of activated macrophages. The first cellular changes are visible within 24 hours after induction of a hemodynamically relevant stenosis, which indicates that the molecular response occurs very quickly [8–10].

Arteriogenesis (collateral growth) thus is an entity of its own that has to be clearly distinguished from angiogenesis, the sprouting of capillaries [8–11].

In addition to circulating cells recent studies suggest an important role of tissue resident vascular progenitor cells during postnatal neovascularization [10, 12–15]. CD133^+^ pluripotent cells were found in liver, intestine, muscles, and exocrine glands [14]. In cardiac tissue, cardiovascular progenitor cells were found to be developed from a Flk-1 positive stem cell population [16]. We previously demonstrated that collateral growth was associated with proliferation and differentiation of resident perivascular cells suggesting that tissue resident cells play a dominant role during adult neovascularization [17].

In this study we embarked on a search for regulatory elements particularly of collateral growth on the cell membranous level that might explain the rapid adaptation of vascular cells during the initial phase of collateral growth. We utilized a model of arteriogenesis in the rat hindlimb that allowed us to identify and isolate a single collateral vessel early after induction of collateral growth [8]. This collateral artery was used for all biochemical and histological analyses except for those experiments where isolated vascular resident progenitor cells in culture were utilized as described in the Materials and Methods and Results sections. Based upon the generation of monoclonal antibodies against membrane preparations of this collateral vessel and immunohistochemical screening for collateral selectivity we generated a monoclonal antibody (CTA 157-2) that bound to the cell membrane of cells in the endothelial lining of growing collateral vessels as well as of isolated vascular resident endothelial progenitor cells (VR-EPCs) that were recently characterized by our group [18]. Interestingly CTA 157-2 was found to bind and activate extracellular proteasome. Furthermore the antibody reduced proliferation of VR-EPCs* in vitro* and collateral growth* in vivo* pointing towards an important functional role of cell surface bound proteasome during vascular resident endothelial progenitor cell proliferation and collateral growth.

## 2. Materials and Methods

All investigations conformed to the Guide for the Care and Use of Laboratory Animals published by the US National Institutes of Health (NIH Publication number 85-23, revised 1996) and Section 8 of the German Law for the Protection of Animals. Except for the immunization all* in vivo *experiments were performed on male Sprague Dawley rats (weight 200 g, Charles River Laboratories, Germany) under general anesthesia as previously described in detail [8].

### 2.1. Generation of Collateral Prone Antibody CTA 157-2

For collateral membrane proteins collection, 25 male SD rats were used. The right femoral artery of SD rats was ligated under general anesthesia as previously described [8]. Animals were killed twelve hours after femoral artery occlusion. Collateral arteries were identified using postmortem angiographies as previously described [8], excised, and shock frozen. Our previous experiments had shown that no degradation of mRNA or proteins occurred during this procedure. Collateral arteries were then homogenized in membrane braking buffer containing 50 mmol Tris HCl pH 7,5, 0,32 M Sucrose, 3 mM MgCl_2_, 10 mM iodoacetamide, 0.02% Na Azide, 5 mmol EDTA, and proteinase inhibitors (all reagents: Sigma; St. Louis, USA). They were centrifuged twice at 1000 g and 4°C. Protein concentration of supernatant was determined and centrifuged at 100,000 g, 4°C for 1 h.

The antibody was generated according to standard protocols originally described by the Nobel laureates Kohler and Milstein [19]. Three C57/B16 (weight 20 g, Charles River Laboratories, Germany) mice were used for immunization. Anesthesia was induced by intraperitoneal injections of Ketamine (100 mg/kg body wt, Atarost) and 2% Xylazine (5 mg/kg body wt, Bayer). C57/B16 mice were immunized with membrane preparations of collateral arteries in TiterMax adjuvant (Sigma; St. Louis, USA) by 3 intraperitoneal injections at 2 weekly intervals. Two days after a fourth terminal boost, spleens were harvested and lymphocytes were fused with SP2/O mouse myeloma cells as previously described [20]. We screened and selected clones using cryosections of activated collateral arteries and control vessels.

For* in vitro* and* in vivo* investigations, the antibody was purified using Affinity Pak Immobilized Protein-L Columns (Pierce, Rockford, USA) according to manufacturer's instructions.

Immunohistochemistry staining of proliferating collaterals and control nonproliferating vessels was performed as described previously [8]. For double staining, CTA 157-2 was linked to NHS-Rhodamine (Pierce, Rockford, USA) using protocols provided by the manufacturers.

### 2.2. Isolation and Culture of Adult Vascular Resident Cells

Rat vascular resident cells were isolated as described before [18]. Briefly, rat hearts were perfused* ex vivo* with Krebs-Ringer buffer containing 0.06% collagenase. Cardiac microvasculature cells were then collected from the recirculation medium. Cells were grown under standard cell culture conditions using Dulbecco's Modified Eagle Medium (DMEM) supplemented with 10% Fetal Calf Serum (FCS), glutamine, and antibiotics. Confluent monolayers were split routinely 1 : 4 after washing with PBS and treatment with trypsin-EDTA. All reagents were purchased from Invitrogen (Karlsruhe, Germany).

### 2.3. Clonogenic Assay

After sorting, CTA 157-2 positive cells were cultured with DMEM + 10% FCS. When reaching 80% confluence, cells were detached and sorted by DAKO Cytomation MoFlo High Speed Cell Sorter (DAKO, Denmark) and single cells were placed in a 96-well plate. Cell growth was examined and counted every day by Nikon eclipse TS100 microscope (Nikon, Japan). After 7 days, wells with colony forming units were selected and subcultured for further studies.

### 2.4. Staining for Immunohistochemistry and FACS Analysis

#### 2.4.1. Antibodies

Primary antibodies were anti-vimentin (V9) (Dianova, Hamburg, Germany); anti-PI3-kinase p85 (Upstate, New York, USA); anti-paxillin (C-18) polyclonal goat antibody (Santa Cruz; CA, USA); anti-plectin (C-20) polyclonal goat antibody (Santa Cruz; CA, USA); anti-vinculin polyclonal goat antibody (Santa Cruz; CA, USA); mouse monoclonal anti-p 27^kip1^ antibody (Abcam, Cambridge, UK); and polyclonal rabbit anti-bovine 20S proteasome antibody (Zymed, San Francisco, CA, USA).

Secondary staining was performed with FITC-coupled goat anti-mouse or donkey anti-goat antibody (Dianova, Hamburg, Germany). For light microscopic analysis secondary staining was performed with a peroxidase coupled goat anti-rabbit or goat anti-mouse antibody (Dianova, Hamburg, Germany). Revelation was performed using DAB (3,3′-diaminobenzidine). Counterstaining for light microscopic images was performed with hematoxylin.

#### 2.4.2. Immunohistochemistry

Immunohistochemistry on tissue sections was performed as described previously [8]. For double labelling in immunohistochemistry and flow cytometric analysis CTA 157-2 was linked to NHS-Rhodamine (Pierce, Rockford, IL) or NHS-Dye 680 (MoBiTec), respectively, using protocols provided by the manufacturers. After fixing the cells either with ethanol 70% at pH 2 or with acetone : methanol 1 : 1 followed by blocking with 1% FCS in PBS they were treated with primary antibodies for 45 minutes at 37°C. Secondary antibodies were incubated for 45 minutes at 37°C. Nuclei were stained with 1 *μ*g/mL Hoechst 33342 (Cambrex, Verviers, Belgium). Double staining was analyzed with a confocal laser scanning microscope (Zeiss, Oberkochen, Germany). Staining with labeled CTA 157-2 was performed either before or after permeabilization as indicated.

### 2.5. Chromatographic Purification of VR-EPCs Membrane Proteins

Membrane fractions were separated via ion-exchange chromatography on a Source 30 Q column (running buffer: 50 mmol Bis-Tris pH 6.5, 0.25% Triton; elution buffer: 50 mmol Bis-Tris pH 6.5, 0.25% Triton, 0.2, 0.3, 0.5, and 2 M NaCl; Pharmacia Biotech, Freiburg, Germany). Fractions were collected and tested for antibody binding in a nondenaturing slot-blot. Positive fractions were further separated via gel filtration using a Superdex Tricorn 200 HR 10/30 column (running buffer: 20 mmol Bis-Tris pH 6.5, 60 mmol NaCl; Amersham Biosciences, Uppsala, Sweden). Positive fractions were further separated via high liquid chromatography on a Mini Q Tricorn column (running buffer: 20 mmol Bis-Tris, pH 6.5 50 mmol NaCl; elution buffer 50 mmol Bis-Tris, pH 6.5, linear gradient of 2 M NaCl from 0 to 50%; Pharmacia Biotech, Freiburg, Germany). Positive fractions were run on 10% SDS page.

### 2.6. Mass Spectrometry (MS)

Protein spots were cut out, the proteins were reduced with DTT, the cysteine residues were modified with iodoacetamide, and the protein in-gel was digested with trypsin (modified trypsin, Promega). After digestion the peptides were extracted from the gel, desalted on a C18mZipTip (Millipore), eluted with 1 *μ*L 60% methanol/5% formic acid, and analyzed by nanoelectrospray mass spectrometry in a QTOF II instrument (Micromass, Manchester). The MS/MS spectra obtained by collision-induced fragmentation of the peptides were evaluated by the Mascot MS/MS ion search algorithm (Matrix Sciences, London).

### 2.7. Proteasome Activity Assay

20S proteasome activity was measured using the 20S Proteasome Activity Assay Kit according to the protocol provided by the manufacturer (Chemicon International Inc., Temecula, CA). 10 *μ*g of recombinant 20S proteasome was treated with different concentrations of CTA 157-2 and IgM control (0, 4, 8, 10, 15, and 20 mg/mL, resp.) as well as with lactacystin (0, 0.75, 1.5, 3, and 6 *μ*g/mL, resp.) for 15 minutes at room temperature before adding the proteasome substrate (Suc-LLVY-AMC). Afterwards samples were incubated for 2 h at 60°C and the fluorescence was recorded using a 355/460 filter set in a fluorometer. Proteasome activity as total amount of proteasome substrate cleaved was calculated from a standard curve and related to the zero value for comparison between the experiments. A total of 8 independent experiments were performed.

### 2.8. Immunoprecipitation and Staining with a Second Polyclonal Rabbit Anti-Bovine 20S Proteasome Antibody

Purified CTA 157-2 (5–10 mg/mL) antibody was dissolved in 0.1 M NaHCO_3_ buffer containing 0.5 M NaCl, pH 8.4 (binding buffer 1). Cyanogen-bromide activated resin (Sigma Chemicals, St. Louis, MO) was washed and swollen in cold 1 mM HCl for at least 30 minutes and washed in distilled water followed by washing with the coupling buffer and transferred to the solution with the antibody as described by the manufacturer. Antibody and gel were mixed at 4°C overnight. Unreacted antibody was washed away with binding buffer and unreacted groups were blocked with 0.2% glycine pH 0.8 for 2 hours at room temperature. The blocking solution was removed by washing with the binding buffer 1 and then with acetate buffer (0.1 M. pH 4, containing NaCl 0.5 M). The CTA 157-2 coupled sepharose beads were equilibrated with TNT buffer containing Tris 1 M pH 7.4, NaCl 5 M, Triton 10%, and proteinase inhibitors. CTA 157-2 positive cells were also dissolved in TNT buffer and preincubated with uncoupled cyanogen-bromide beads before incubation with the CTA 157-2 coupled sepharose beads at 4°C overnight. After incubation the beads were centrifuged and washed three times with TNT buffer before subjecting them to gel electrophoreses and western blotting. After blocking, blots were incubated with a polyclonal rabbit anti-bovine 20S proteasome antibody (Zymed, San Francisco, CA, USA). Revelation with ECL was performed after incubation with a peroxidase coupled goat anti-rabbit antibody as described above. For control experiments a control IgM antibody was linked to the resin. All experiments were performed on cellular extracts of CTA 157-2 positive and CTA 157-2 negative cells.

### 2.9. Western Blots

VR-EPCs were homogenized in lysis buffer (Cell Signaling, Massachusetts, USA) containing a cocktail of protease inhibitors, and the protein concentration was determined by Lowry assay (BioRad, Germany). Samples were separated in 10% PAGE-SDS gels. After transfer to nitrocellulose membranes, samples were blocked in 5% albumin and incubated with primary antibodies for 2 hours at room temperature. After 4 washing steps with PBS, membranes were incubated with peroxidase-conjugated secondary antibodies (Dianova, Hamburg, Germany). Finally, detection was performed using a chemiluminescence system (GE Healthcare, Germany).

### 2.10. Flow Cytometric Analyses

For flow cytometric analysis of CTA 157-2 the antibody was linked to NHS-Dye 680 (MoBiTec, Göttingen, Germany) using protocols provided by the manufacturers. VR-EPCs were harvested, washed with PBS, and incubated for one hour with the antibody. Finally, cells were fixed and analyzed with a flow cytometer (FACSCalibur, Becton Dickinson, USA). For quantification we used DIVA software (Becton Dickinson, USA).

### 2.11. *In Vitro* and* In Vivo* Evaluation of VR-EPCs Functions Using CTA 157-2 Monoclonal Antibody

CTA 157-2 and control IgM antibody (both 10 *μ*g/mL) were added into the culture medium of subconfluent VR-EPCs. After 2 days, assessment of* in vitro* proliferation was performed as described previously using the BrdU Flow Kit (PharMingen GmbH, Germany). After incubation with Bromodeoxyuridine (BrdU, 0.3 mg/mL) for 2 hours, the number of positive BrdU cells was counted by flow cytometry (FACSCalibur, Becton Dickinson, USA) as described previously [21]. A total of 5 independent experiments were performed. For* in vivo* assay, femoral artery occlusions were performed. CTA 157-2 (0.1 mg/mL at 10 *μ*L/h) as well as the carrier alone was infused directly into the collateral circulation via osmotic minipump (ALZET, USA). After 7 days, proliferation of the collateral artery was detected by BrdU labeling and detection Kit 2 (Roche Diagnostics, Germany) as described previously [8], and the proliferation index was calculated as the number of BrdU-positive nuclei to the total number of nuclei inside the vessel wall (*n* = 6 per group). Postmortem angiographies (*n* = 6 per group) were obtained 7 days after femoral artery occlusion as described previously using postmortem perfusion of the distal hindlimb with a barium based contrast agent and exposition on single paper wrapped films (X-OMAT MA 13 × 18 cm, Kodak, France) in an X-ray chamber (Faxitron X-ray cooperation, Model 43855D, USA).

### 2.12. Collateral Proliferation

Collateral proliferation (*n* = 6 per group) was detected 7 days after cell administration as described previously with BrdU labeling and detection Kit 2 (Roche Diagnostics, Germany).

### 2.13. Determination of Collateral Dependent Conductance

Measurement of total collateral dependent conductance was performed one week after femoral artery occlusion in the anesthetized animal. In order to determine collateral conductance a method previously established and validated for the rabbit hindlimb was adapted to investigate the functionality of collateral vessels in the rat hindlimb at maximal vasodilatation [22]. We determined total collateral dependent conductance instead of pure collateral conductance because due to animal size placement of a pressure transducer distal to the collateral circulation distorts pressure and blood flow measurements in the rat hindlimb. Furthermore it has been shown that total collateral dependent conductance reflects collateral conductance within the first week after femoral artery occlusion [22, 23]. Systemic blood pressure, identical to blood pressure, was measured invasively via a catheter placed into the carotid artery and connected to a pressure transducer (FME TBD1220, Ser. number 2955; För Medical Instruments). Venous blood pressures were determined via a 1.4F Millar ultraminiature pressure only catheter (För Medical Instruments) that was inserted into the jugular vein and advanced to the vena cava superior. Collateral blood flow was determined via an ultrasonic flow probe (Transonic flow probe, V-Series; style: 0.7 mm, Probe: 0.5 PSB 263, Transonic Systems Inc., NY, USA) placed around the collateral stem region (common iliac artery) and connected to the respective flow meter (Transducer Ts 420, transit-time perivascular flowmeter Transonic Systems Inc., NY, USA). Measurements were performed under maximal vasodilatation using adenosine at increasing concentrations ranging from 15 to 300 *μ*g/Kg per minute via a catheter inserted into the left carotid artery and advanced to the bifurcation as described previously. All measurements were recorded online on a computer using the MacLab interface (PowerLab; UmacLab). Total collateral dependent conductance was calculated as described previously dividing collateral blood flow by the arteriovenous pressure difference [22].

### 2.14. Statistical Analysis

All cell culture and biochemical analyses were repeated in at least 3 independent experiments. Data are shown as mean ± SEM. Statistical comparisons between 2 groups were performed with two-tailed Student's *t*-test. Multiple comparisons between groups were applied when necessary with ANOVA followed by post hoc analysis. Differences among means were considered significant when *P* < 0.05.

## 3. Results

### 3.1. Monoclonal Collateral Targeting IgM Antibody (CTA) 157-2 Stains Particularly the Endothelium of Proliferating Collateral Arteries

**Figure 1:**
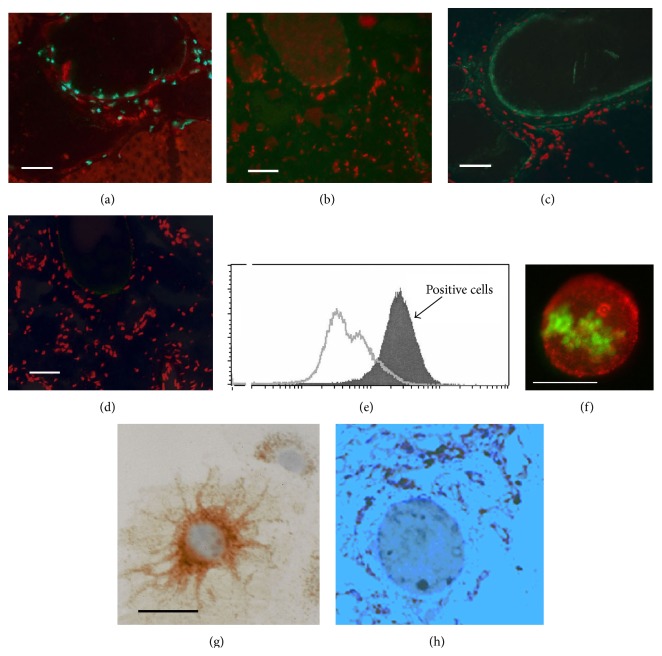
mAb CTA 157-2 staining of the endothelial lining of proliferating collateral vessels but not of control vessels ((a) and (c) parallel sections of proliferating collateral vessel; (a) BrdU staining (green fluorescence), (c) mAb CTA 157-2 staining (green fluorescence); (b) and (d) parallel sections of control vessel; (b) absence of BrdU staining (green fluorescence), (d) absence of mAb CTA 157-2 staining (green fluorescence); scale bar 40 *μ*m). All slides were counterstained with propidium iodide as nuclear staining (red fluorescence). (e) Flow cytometric analysis of nonpermeabilized VR-EPCs after staining with mAb CTA 157-2 demonstrating specific extracellular staining of all cells (filled curve). Open curve shows staining with control antibody. (f) Fluorescence microscopy of BrdU treated cells stained with CTA 157-2 (red fluorescence) before permeabilization followed by permeabilization and staining of BrdU (green fluorescence) demonstrating focal distribution of the antigen on the cell membrane of proliferating VR-EPCs. (g) Light microscopic image of a vascular resident progenitor cell with immunohistochemical staining for total (intra- and extracellular) 20S proteasome (brown color; hematoxylin counterstain: blue): most 20S proteasome is found intracellularly, predominantly, and perinuclearly. (h) Light microscopic image of total 20S proteasome (brown color; hematoxylin counterstain: blue): most total (presumably intracellular) 20S proteasome is found in perinuclear cells.

The immunohistochemical screening of monoclonal antibodies raised against membrane preparations of collateral arteries revealed that collateral targeting IgM antibody (CTA) 157-2 particularly stained the endothelial surfaces of proliferating collateral arteries (Figures 1(a)–1(d)). Other tissues like nerve tissue and other large vessels were stained as well albeit at a much lower level. The staining in these cases appeared to be restricted to the intracellular space. Thus this antibody appeared to be particularly suitable for the identification of novel players in collateral growth and was selected for further analysis.

### 3.2. Extracellular Staining of Vascular Resident Endothelial Progenitor Cell Membranes by CTA 157-2 as Demonstrated via Flow Cytometric Analysis

Using flow cytometric analysis we were able to demonstrate that CTA 157-2 stained an antigen present on the cell membrane of vascular resident endothelial progenitor cells (VR-EPCs), which were previously thoroughly characterized by our group [18] (Figure 1(e)). Fluorescence microscopy of these cells loaded with BrdU and stained with CTA 157-2 before permeabilization followed by permeabilization and staining of BrdU demonstrated a focal distribution of the antigen on proliferating cells (Figure 1(f)). This staining pattern contrasted with staining for total 20S proteasome that showed predominantly an intracellular perinuclear distribution as described previously [4] (Figure 1(g)). In histological section of collateral vessels this predominantly intracellular proteasome was mainly found in perivascular cells, presumably macrophages that are known to accumulate during collateral growth (Figure 1(h)).

### 3.3. CTA 157-2 Stains a 700 kDa Antigen Identified as Proteasome after Chromatographic Purification of Endothelial Membrane Fractions

**Figure 2:**
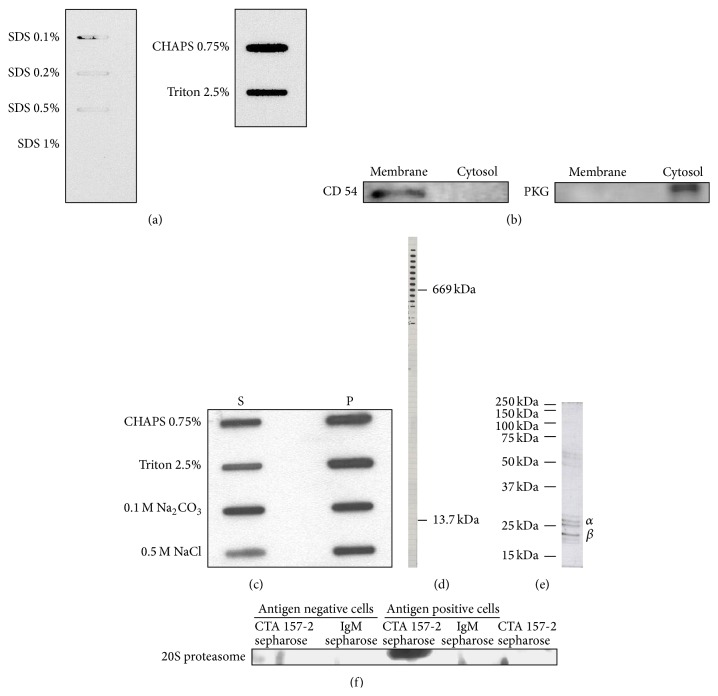
(a) Slot blot analysis of binding capacity of CTA 157-2 with different concentrations of SDS, CHAPS, and Triton. (b) Confirmation of purity of membrane fractions with anti-CD 54 antibody and cytosolic fractions with anti-PKG antibody. (c) Solubility of VR-EPC membrane fraction in different detergents (S: soluble phase; P: precipitate). (d) CTA 157-2 staining of different fractions collected after gel chromatography of cell membrane preparations along with stained marker proteins of defined sizes reveals that CTA 157-2 stains a protein complex of 700 kDa. (e) This complex separates on conventional SDS page into several subunits identified as 26 kD *α*7 and 21 kD *β*3 proteasome in mass spectrometry. (f) Specific staining of 20S proteasome after immunoprecipitation with CTA 157-2 of VR-EPC extracts (antigen positive cells). No staining after immunoprecipitation of antigen negative rat heart microvascular cells (antigen negative cells). No staining after immunoprecipitation with IgM control and of CTA 157-2 sepharose beads.

In our attempt to identify the antigen stained by CTA 157-2 we had to learn that SDS even at lower concentration inhibited binding of CTA 157-2 to this antigen suggesting that this antibody detects a tertiary protein structure (Figure 2(a)). For this reason classical western blots could not be performed and classical methods of protein identification like screening of c-DNA expression libraries were deemed to fail. The purity of the membrane preparation was confirmed after staining with antibodies against CD 54, a membrane bound cell adhesion molecule, and soluble PKG a cytosolic protein on western blot. Only the membrane fractions expressed CD 54 whereas PKG was only found in the cytosolic fraction (Figure 2(b)). We tested the solubility of the complex in membrane fractions using different detergents and were able to demonstrate that part of the complex was solubilized already with NaCl. Only nonionic or zwitterionic detergents like CHAPS, Triton, or Tris, which leave tertiary structures of membrane proteins intact, allowed CTA 157-2 to detect the antigen indicating that the antigen-binding site represents a tertiary protein structure possibly composed of several protein subunits (Figure 2(c)). This also indicated a fairly strong attachment of the antigen detected by CTA 157-2 to the membrane fraction. The antigen was finally identified after chromatographic purification of membrane preparations of RHE A cells. Gel filtration of membrane fractions revealed that the antigen had a size around 700 kDa (Figure 2(d)). After further purification using ion exchange chromatography the antigen separated into several subunits on SDS page that were identified as 26 kDa *α*7 and 21 kDa *β*3 subunits of 20S proteasome using mass spectrometry (Figure 2(e)). These were the only rat proteins sequenced from the final CTA 157-2 positive fraction indicating that the purification strategy was successful.

### 3.4. Immunoprecipitates Generated with CTA 157-2 Are Identified as 20S Proteasome with a Polyclonal Rabbit Anti-Bovine 20S Proteasome Antibody on Western Blots

A second independent line of evidence is that CTA 157-2 bound 20S proteasome was generated after immunoprecipitation of cellular extracts with CTA 157-2 covalently linked to sepharase beads. Staining of immunoprecipitated extracts after gel electrophoreses and blotting with a second polyclonal rabbit anti-bovine 20S proteasome antibody revealed a band at 23 kDA corresponding to the 20S proteasome subunits reported to stain with this particular antibody (Figure 2(f)). This band was not detected in cellular extracts after immunoprecipitation with a control IgM and was only visible in immunoprecipitates of VR-EPC cellular extracts but not in cellular extracts from control cells that did not express the antigen in FACS analysis (Figure 2(f)).

### 3.5. CTA 157-2 Binds to Purified 20S Proteasome and Leads to Its Activation

**Figure 3:**
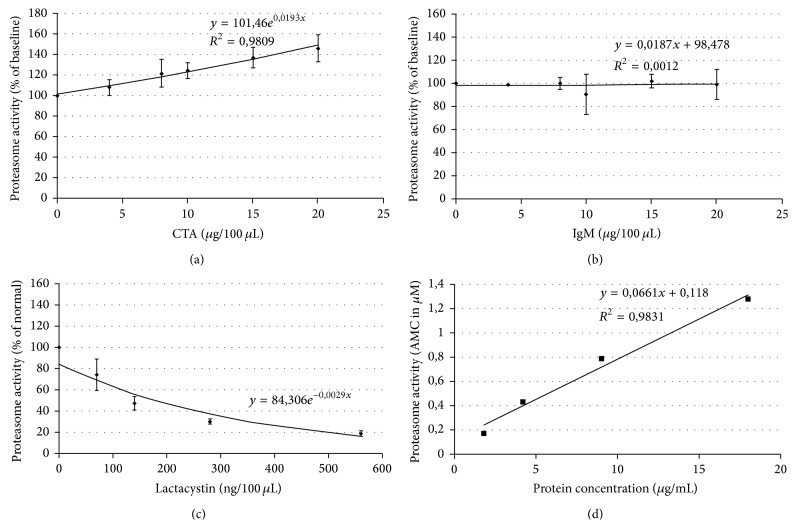
(a) CTA 157-2 specifically activates 20S proteasome and (b) control IgM antibody is without effect. (c) Proteasome inhibitor lactacystin reduces proteasome activity as expected. (d) Activation of the proteasome is purified membrane preparations with CTA 157-2 treatment.

We furthermore revealed a functional impact of CTA 157-2 on the proteasome when testing different concentrations of CTA on AMC cleavage from LLVY by 20S proteasome. CTA 157-2 specifically and concentration dependently activated reproducibly 20S proteasome (Figure 3(a)). We were able to exclude that this activation was due to unspecific effects of IgM (Figure 3(b)). Functionality of the assay was confirmed with the known proteasome inhibitor lactacystin (Figure 3(c)).

### 3.6. CTA 157-2 Elicits Proteasome Activity in Purified Membrane Fractions of VR-EPC

In order to confirm that proteasome activity is present in cell membranes we tested the effect of different membrane protein concentrations on AMC cleavage from LLVY with and without CTA 157-2 stimulation. In accordance with previous reports we were not able to detect any significant proteasome activity without stimulation. With CTA 157-2 stimulation, however, we were able to detect a concentration dependent proteasome activity of purified cell membranes (Figure 3(d)).

### 3.7. Proteasomal Activation via mAb CTA 157-2 Inhibits Concentration Dependently VR-EPC Proliferation* In Vitro*

**Figure 4:**
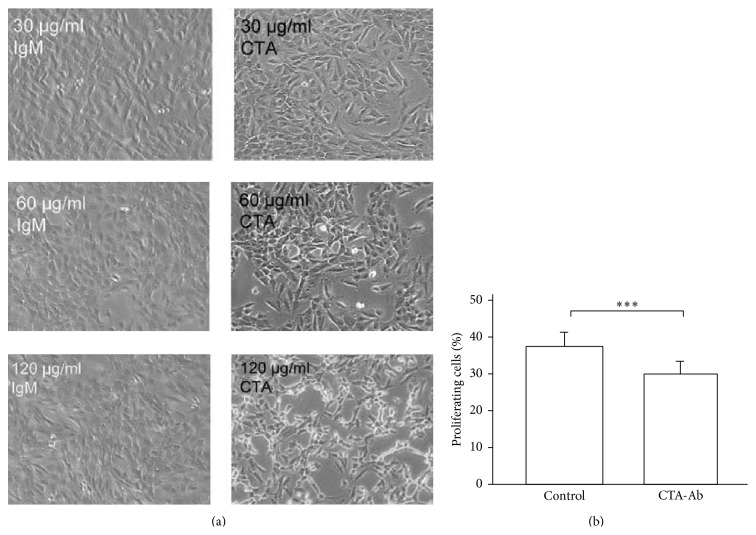
Concentration dependent decrease of VR-EPC proliferation by treatment with CTA157-2: (a) microscopic images after addition of control IgM and CTA 157-2 at different concentrations; (b) quantitative comparison of VR-EPC proliferation after addition of control IgM and CTA 157-2 as determined by BrdU incorporation; 5 experiments; ^*∗∗∗*^ *P* < 0.001.

*In vitro* CTA 157-2 reduced VR-EPCs proliferation concentration dependently (Figures 4(a) and 4(b): 37.2% ± 4.4% (IgM control) versus 29.7% ± 3.6% (CTA 157-2), ^*∗∗∗*^ *P* < 0.001 at 120 *μ*g/mL).

### 3.8. Proteasomal Activation via mAb CTA 157-2 Inhibits Collateral Proliferation* In Vivo*

**Figure 5:**
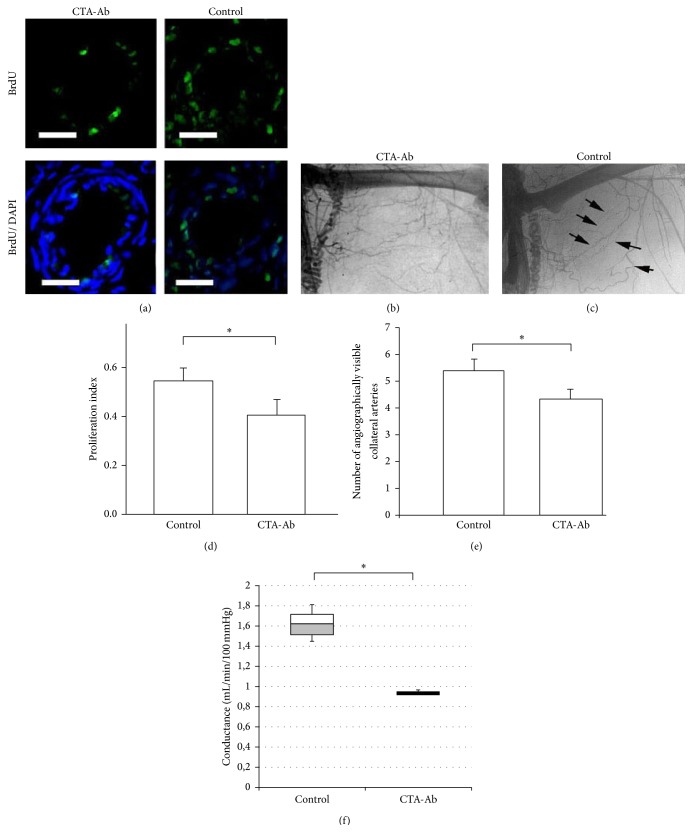
Decreased proliferation, number of collateral vessels, and total collateral conductance after* in vivo* injection of CTA 157-2 as compared to injection of control IgM: (a) BrdU incorporating nuclei (green/FITC) in relation to total nuclei (blue staining/DAPI) in collateral vessels of CTA 157-2 treated and control animals, (b) postmortem angiography of animal treated with CTA157-2, and (c) postmortem angiography of control animal (black arrows: collateral vessels). (d) Quantitative comparison of proliferation index (scale bar represents 100 *μ*m; 6 animals per group, ^*∗*^ *P* < 0.05). (e) Quantification of visible collateral vessels using stereoscopic analysis (6 animals per group; ^*∗*^ *P* < 0.05). (f) Total collateral conductance in animals treated with CTA 157-2 as compared to control (3 animals per group; ^*∗*^ *P* < 0.05).

In order to investigate the impact of proteasomal activation on collateral growth we used the CTA 157-2 antibody in a collateral artery growth model previously described by our group [8, 17, 23]. In contrast to injection of an IgM control antibody local infusion of CTA 157-2 antibody reduced collateral proliferation and formation of collateral arteries in postmortem angiographies (Figures 5(a) and 5(d): proliferative index CTA 157-2 versus control IgM: 0.40 ± 0.14 versus 0.54 ± 0.17, ^*∗*^ *P* < 0.05; Figures 5(b), 5(c), and 5(e): number of visible collateral vessels CTA 157-2 versus control IgM 4.3 ± 0.7 versus 5.3 ± 0.4; ^*∗*^ *P* < 0.05). Furthermore we were able to demonstrate on a functional level infusion of CTA 157-2 reduced total collateral conductance after femoral artery occlusion as compared to control (Figure 5(f): box plot of total collateral conductance in mL/min/100 mmHg: CTA 157-2 versus control IgM: 0.93 ± 0.02 mL/min/100 mmHg versus 1,61 ± 0.2 mL/min/100 mmHg, ^*∗*^ *P* < 0.05).

## 4. Discussion

In this study we demonstrate that transmembranous proteasomal activation controls vascular endothelial progenitor cell proliferation and collateral growth. The primary tool for our investigations was the monoclonal antibody CTA 157-2 that was generated against membrane preparations of growing collateral vessels in an attempt to identify novel membrane bound players in collateral growth. The collateral prone antibody CTA 157-2 was chosen because it predominantly stained the endothelial lining of proliferating collateral vessels, bound 100% of VR-EPCs in flow cytometric analysis, and was shown to significantly inhibit their proliferation concentration dependently* in vitro*. We furthermore were able to demonstrate that infusion of this antibody significantly inhibited collateral growth in contrast to infusion of an IgM control antibody.

Despite the challenging nature of the antibody and the antigen bound by this antibody we managed to obtain four lines of evidence that CTA 157-2 binds to cell membranes and activates the proteasome. A number of activators and inhibitors have been shown to regulate 20S proteasome* in vitro* [4]. Some activators like PA 28 bind to the end of the barrel shaped proteasome complex and stimulate peptidase activity. It appears possible that CTA 157-2 also binds to the ends of 20S proteasome and simulates activator functions [4].

More importantly we were able to demonstrate that the binding site of CTA 157-2 is exposed on the extracellular surface of vascular cell membranes via flow cytometric and immunohistochemical analysis of impermeabilized VR-EPCs. While the major part of the proteasome is located within the nuclear and cytoplasmic compartment it has long been recognized that the proteasome is also associated with cell membranes [24]. Lipopolysaccharide (LPS) components of Gram-negative bacteria have been shown to specifically bind to 20S proteasomes attached to the cell membrane of murine macrophages [25]. The presence of proteasomes at the surface of cell membranes was furthermore reported in human T and B lymphocytes [26]. Proteolytic active 20S proteasome was also detected in lung epithelial lining fluid of patients with acute respiratory distress syndrome [27]. Recently it was shown that functionally active 20S proteasome can be exported from activated immune cells by way of microparticles [28]. Although extracellular proteasome is known to be elevated in a number of inflammatory processes reports of its functional role during inflammation are scarce.

Inflammatory processes are strongly connected to arteriogenesis. In particular macrophage accumulation and activation have been shown to play a very important role in collateral growth [8–10, 17, 29]. A number of reports demonstrate that proteasomal activity is regulated by paracrine signals. It has, for example, been shown that cell attachment to extracellular matrices induces proteasomal degradation of the cyclin-dependent kinase 2 inhibitor p 21 [30]. To our knowledge, however, this is the first report demonstrating a functional role of extracellular 20S proteasome on progenitor cell function and vascular growth. Previous studies demonstrated that inhibition of the proteasome reduces angiogenesis and the growth of vascular cells, which at first sight appears to contradict our findings [31, 32]. However these studies investigated the effect of the proteasome on angiogenesis, the sprouting of capillaries, which considerably differs in its mechanisms from arteriogenesis, and the growth of collateral arteries from preexisting arteriolar anastomoses [10, 11]. Furthermore these studies primarily targeted the proteasome in nuclear and cytoplasmic compartments using different inhibitors and approaches whereas mAb CTA 157-2 only binds to the extracellular compartment* in vitro* and* in vivo*. For the same reason it appears counterintuitive to use proteasome inhibitors like lactacystin to test our hypothesis in cell culture or in* in vivo* experiments because it targets the proteasome in a different compartment and in different cells. To our knowledge there are no other agents that target specifically extracellular, respectively, transmembraneous proteasome apart from mAB CTA 157-2.

In this context it is interesting to note that the SOCS box protein 5 (asb5) that acts as a bridge between specific substrate-binding domains and E3 ubiquitin protein ligases has previously been shown to be upregulated in growing collateral vessels [33]. However a functional impact of this part of the ubiquitin-proteasome system on either vascular cell proliferation or collateral growth has not been demonstrated until now. Furthermore it was shown that adenoviral transfection of PR39, a protein that enhances hypoxia-inducible factor-1 alpha- (HIF-1 alpha-) dependent gene expression by selectively inhibiting proteasome degradation of this transcription factor, improves blood flow and myocardial function in a pig model of chronic myocardial ischemia by enhancing collateral formation [7].

## 5. Conclusion

Our study adds further evidence to the notion that the ubiquitin-proteasome system plays a decisive role in regulating collateral growth and demonstrates that extracellular 20S proteasome not only is released from cells but also plays a functional role in physiological processes.
